# Probiotic Potential and Cholesterol-Lowering Capabilities of Bacterial Strains Isolated from *Pericarpium Citri Reticulatae* ‘Chachiensis’

**DOI:** 10.3390/microorganisms9061224

**Published:** 2021-06-04

**Authors:** Qianxian He, Jingyu Li, Yongkai Ma, Qi Chen, Gu Chen

**Affiliations:** School of Food Science and Engineering, South China University of Technology, Guangzhou 510640, China; QianxianHe@scut.edu.cn (Q.H.); JingyuLi@scut.edu.cn (J.L.); Mayongkai@scut.edu.cn (Y.M.); chenqi@scut.edu.cn (Q.C.)

**Keywords:** *Pericarpium Citri Reticulatae* ‘Chachiensis’, probiotic bacteria, *Bacillus*, *Lactobacillus*, cholesterol-lowering potential

## Abstract

*Pericarpium Citri Reticulatae* ‘Chachiensis’ (PCR-Chachiensis), the pericarps of *Citri Reticulatae* Blanco cv. Chachiensis, is a food condiment and traditional medicine in southeast and eastern Asia. Its rich and various bacterial community awaits exploration. The present study is the first report on probiotic screening and characterization of bacteria from PCR-Chachiensis. Based on 64 culturable bacterial isolates, 8 strains were screened out to have great survival in the simulated gastrointestinal stressful condition, being nonhemolytic and without biogenic amine formation. They were identified by 16S rRNA gene sequencing as two *Bacillus*, three *Lactobacillus*, and three strains from Bacillales. Their probiotic properties, cholesterol-lowering potential and carbohydrate utilization capability were further investigated. Though these eight strains all displayed distinct cholesterol removal potential, *Bacillus licheniformis* N17-02 showed both remarkable cholesterol removal capability and presence of bile salt hydrolase gene, as well as possessing most of the desirable probiotic attributes. Thus, it could be a good probiotic candidate with hypocholesterolemic potential. *Bacillus megaterium* N17-12 displayed the widest carbohydrate utilization profile and the strongest antimicrobial activity. Hence, it was promising to be used as a probiotic in a host and as a fermentation starter in fermented food or feed.

## 1. Introduction

*Pericarpium Citri Reticulatae* (in Chinese *Chen Pi*) is the dried and aged pericarps of *Citrus reticulata* Blanco and its cultivars. Other than being a food condiment used widely in cuisine and beverages for hundreds of years in southeast and eastern Asia, it is also a traditional medicine to cure chronic indigestion and respiratory diseases [1]. Extensive studies have been reported on its phytochemistry composition, especially phenolic composition [1]. Recently, bioactivities, such as antioxidant, antifungal and antiviral activities, were reported to boost its health benefits [2,3,4,5]. For example, the neuroprotective effects of the flavonoids and the antiviral activity of the polymethoxylated flavones [3,5]. *Pericarpium Citri Reticulatae* ‘Chachiensis’ (PCR-Chachiensis, in Chinese *Guang Chen Pi*), is the aged pericarps of *Citri Reticulatae* Blanco cv. Chachiensis and it has been proven by long-term practice as the premium *Pericarpium Citri Reticulatae* [1,2,6,7,8]. Our previous study has revealed the central bacterial community in PCR-Chachiensis for the first time through amplicon sequencing, and identified *Bacillus* as the most dominant genus [6]. These special bacterial communities might contribute to the superior quality of PCR-Chachiensis.

Probiotics are defined as living microorganisms that confer various health benefits upon the host when ingested in adequate amounts [9]. Probiotics are gaining popularity worldwide and there is an increasing market for functional food and feed calls for novel probiotics [10,11]. Among the known probiotics, lactic acid bacteria (LAB) (e.g., *Lactococcus* and *Lactobacillus*) and *Bifidobacterium* have a long utilization history, while new probiotics such as yeast and *Bacillus* and more specific LAB strains are being continuously identified [9,11,12,13,14,15]. In a recent report, the probiotic value of *Bacillus* was underscored due to its elimination of dangerous pathogen *Staphylococcus aureus* via interference of *S*. *aureus* quorum sensing [14]. Their human data indicate that probiotic *Bacillus* can comprehensively abolish intestinal as well as nasal *S*. *aureus* colonization. In addition to their diversified functional properties, *Bacillus* strains have relatively higher resistance to food matrix, food processing and the simulated gastrointestinal tract conditions than the *Lactobacillus* strains evaluated [16,17], thus rendering them as good candidates for processed functional food product.

Other than healthy humans, traditional natural fermented food is another good source to identify new probiotics due to its long history of safe consumption [11,18,19]. Since abundant and various bacteria OTU (operational taxonomic units) including *Bacillus* have been identified in PCR-Chachiensis through culture-independent methods, we further investigated the probiotic potential of the culturable bacterial strains in PCR-Chachiensis. Following the probiotics selection criteria established by the World Health Organization (WHO), strains isolated from PCR-Chachiensis were subjected to assays of host-associated stress resistance, adhesion ability, antimicrobial activity and safety assessment. Carbon source utilization and cholesterol-lowering capabilities were further investigated to explore their functional properties. To the best of our knowledge, our study is the first report on the probiotic characterization of bacterial isolates from PCR-Chachiensis.

## 2. Materials and Methods

### 2.1. Isolation and Identification of Bacterial Strains in PCR-Chachiensis

The PCR-Chachiensis samples (Figure S1) used in this study originated from different orchards of Xinhui District, Jiangmen City in Guangdong Province of China and they were authenticated by Yonghe Chen form Xiangyi Chenpi Company. Samples of PCR-Chachiensis were first mixed with the liquid medium, respectively MRS (de Man, Ragosa, Sharpe), Elliker’s medium and LB (Luria–Bertani) medium at 37 °C for 12 h with shaking. The mixtures were spread onto plates containing the same medium and incubated at 37 °C for 24 h. Colonies with different morphologies were selected and cultured to obtain a single colony for further screening. All selected strains were stored in equivalent mixed solutions of 50% (*v/v*) glycerol and medium at −80 °C.

Genomic DNA was isolated from each pure culture of isolated strains using the bacterial DNA extraction kit (Dongsheng Biotech, Guangzhou, China) and amplified using polymerase chain reaction with universal primers (27F-5′-AGAGTTTGATCCTGGCTCAG-3′ and 1492R-5′-GGTTACCTTGTTACGACTT-3′). The amplified 16S rDNA sequences were identified by commercial sequencing service (Tianyihuiyuan Biotechnology Co. Ltd., Guangdong, China). The phylogenetic tree was generated by the neighbor-joining method using MEGA-X 10.1.7 software [20] and was displayed by Evolview (https://evolgenius.info//evolview) (accessed on 28 December 2020).

### 2.2. Hemolytic Test

The strains were streaked or spotted on agar plates containing sheep blood and incubated at 37 °C for 48 h [21]. Strains that had a green zone around the colony (α-hemolysis, partial hydrolysis), or formed a clear zone around the colony (β-hemolysis, complete hydrolysis) failed in the test. Only isolates that did not produce any zone around the colony were designated as no hemolysis.

### 2.3. Resistance To Artificial Gastric Juice and Intestinal Juice

The resistance of strains to gastric acid and intestinal juice was determined following the assay reported previously [22,23] with modifications. Artificial gastric juice contained 0.3% (*w/v*) pepsin from porcine gastric mucosa (Sigma Aldrich, St. Louis, MO, USA) with a pH of 2.5. Artificial intestinal juice was prepared by dissolving 0.3% (*w/v*) bile salt (Sigma Aldrich, St. Louis, MO, USA) and 1 mg/mL trypsin (Biofroxx, Einhausen, Germany) in sterile saline solution and adjusting the pH to 8.0. After overnight cultivation, the strains were precipitated and washed twice with phosphate buffer (PBS, pH = 7.4) and then resuspended in PBS (pH = 2.5 or pH = 8.0). Cell suspension at 1% (*v/v*) was injected into artificial gastric juice and incubated at 37 °C for 4 h or intestinal juice for 6 h respectively. The viable cells before and after incubation were determined by dilution plate counting.

Relative survival ratio (%) = CFU of viable cells survived/CFU of initial viable cells inoculated × 100

### 2.4. Biogenic Amine Formation

Formation of biogenic amines was tested using chromogenic medium as described previously [24]. Bacterial culture was inoculated in one of the four kinds of LB-based screening broth media (pH = 5.0) with 0.006% bromocresol purple: LB control, 0.5% yeast extract, 1% tryptone and 1% sodium chloride; LB-GL, control LB including 0.25% glycerol; LB-GLO, LB-GL including 0.01% ornithine; LB-GLH, LB-GL including 0.01% histidine. After 24 h incubation, the color, pH and absorbance at 590 nm of the supernatant were measured. Using this method, the generation of biogenic amines is indicated as a color change to purple in the precursor amino acid containing medium.

### 2.5. Auto-Aggregation and Hydrophobicity Assay

Auto-aggregation ability was evaluated according to the method described in a previous study [25] with modifications. The overnight culture was centrifuged, washed twice with PBS and then resuspended to OD_600_ of 0.4 ± 0.05. Then, the OD_600_ of upper suspension after incubation at 37 °C for 0, 2, 4, 6 and 24 h was measured.

Auto-aggregation (%) = (1 − A_2,4,6 or 24 h_/A_0 h_) × 100

The measurement of cell surface hydrophobicity followed the method described previously [26] with modifications. The overnight culture was suspended in 0.1 M KNO_3_ (pH 6.2) to 1 × 10^8^ cells ml^−1^ and absorbance measured at 600 nm (A_0_) with a Spectrophotometer UV2300. Then, 1 mL dichloromethane and ethyl acetate were added into 3 mL cell suspension, respectively, and mixed with vortex for 5 min. The OD_600_ of aqueous phase was measured after standing for 15 min (A_1_).

Hydrophobicity (%) = (1 − A_1_/A_0_) × 100

### 2.6. Antimicrobial Activity and Co-Aggregation

The antimicrobial activity of the strains was determined using the microplate assay as described previously [27], against *Escherichia coli* (CMCC 44102), *Salmonella paratyphi* (CMCC 50094), *Staphylococcus aureus* (CMCC 26003) and *Bacillus subtilis* (CMCC 63501) purchased from Guangdong Institute for Drug Control, China. Supernatants of overnight cultures were evaluated in antimicrobial assay in their initial pH or neutralized to pH 6.5. The determination of co-aggregation between isolates and indicator bacteria strains followed the method as described previously [25].

### 2.7. Antibiotic Susceptibility

The selected strains were evaluated for antibiotic susceptibility by disk method [28]. The inhibition zone diameters were measured with a vernier caliper and compared with the criteria mentioned in this reference.

Bile salt hydrolase (BSH) activity was assessed using the method described previously [29,30] with modification. Sodium taurodeoxycholic acid (TDCA) and sodium glycodeoxycholic acid (GDCA) at 0.5% (*w/v*) were used as bile salt substrates. The colonies displaying white precipitates were scored as BSH activity.

To amplify *bsh* gene, degenerate primers were designed by retrieving the corresponding amino acid sequence of BSH from similar strains in NCBI (https://www.ncbi.nlm.nih.gov/) (accessed on 9 September 2020). The degenerate primers used were shown in Table 1. Touchdown PCR was applied in the PCR instrument (Eastwin Scientific Equipments Inc., Suzhou, China) and the PCR amplicons were sequenced. Amino acid sequences of BSHs were aligned through Clustal Omega (http://www.clustal.org/omega/) (accessed on 14 October 2020) [31].

### 2.8. Cholesterol Removal Ability

The cholesterol removal ability was evaluated according to the method described previously [30] with modification. The overnight culture was inoculated at 1 mL/100 mL in LB or MRS medium containing cholesterol at a final concentration of 100 μg/mL. After incubation at 37 °C for 6, 12, 18 and 24 h, the cholesterol left in the supernatant was measured spectrophotometrically via the *o*-phthalaldehyde method and calculated according to the standard curve. The GDCA at 2.8 mM and TDCA at 1.2 mM were used as bile when indicated.

### 2.9. Carbohydrate Utilization Capability

Utilization of seven sugars and two sugar alcohols, including D-glucose, D-xylose, lactose, sucrose, cellobiose, stachyose, D-raffinose, sorbitol and mannitol, were evaluated according to the method described previously [32] with modification. The carbohydrates were prepared into a 10% solution respectively and sterilized through filtration by 0.22 μm Millex^®^-GP (Merck Millipore Ltd., Cork, Ireland). They were used as the main carbon source in the basic MRS (without glucose and meat extract) or LB (without tryptone and yeast extract) medium. The 1% (*v/v*) cell suspension (10^8^ cells mL^−1^) was inoculated into medium containing 2% (*w/v*) carbohydrate solution. The optical densities at 600 nm (OD_600_) were recorded and compared with negative control after incubation at 37 °C for 48 h.

### 2.10. Statistical Analysis

Data were expressed as means ± standard error (SE) of at least three replicates. Analysis of variance (ANOVA) and significance of differences (*p* < 0.05) between the means of parameters in the results were calculated using SPSS 22.0 software (SPSS Inc., Chicago, IL, USA).

## 3. Results and Discussion

### 3.1. Identification of Culturable Bacterial Strains in PCR-Chachiensis

**Figure 1:**
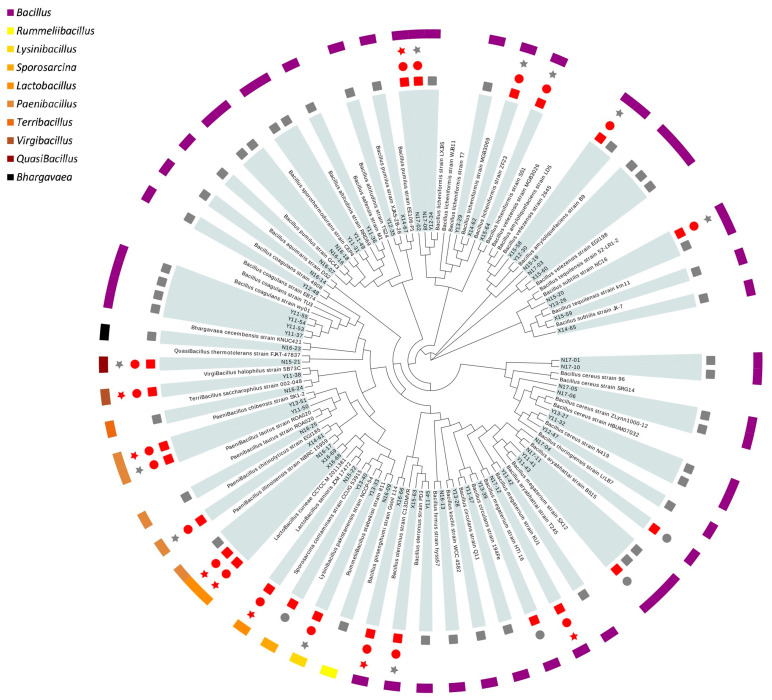
Phylogenetic tree analysis and primary screening of 65 strains isolated from PCR-Chachiensis. The results of hemolytic, gastrointestinal tolerance and the production of amines were respectively represented by symbols from the inside to the outside as square, circle and star. A grey symbol indicates failure and a red one indicates passing the assay.

A total of 64 bacterial strains were isolated from PCR-Chachiensis and their identities were determined based on the phenotypic and genotypic characterization (Figure 1, Table S1). The 16S rDNA sequencing assigned 53 strains in Bacillaceae, among which 49 strains belonging to the genus *Bacillus* and the rest to different genera under Bacillaceae, such as *Lysinibacillus, Terribacillus, Virgibacillus* and *Quasibacillus*. Several strains from genus *Lactobacillus* and *Paenibacillus* were also confirmed, which might open up new opportunities for probiotics.

### 3.2. Hemolytic Activity Test

As displayed in Figure 1, some of the closely related reference *Bacillus* strains such as *B. coagulans*, *B. licheniformis*, *B. subtilis* and *B. megaterium* are currently used as probiotics in clinical trials or commercial products [12], while strains such as *Virgibacillus halophilus*, *Paenibacillus chibensis* and *B. ginsengihumi* have hardly been mentioned. Though *Lactobacillus* strains such as *L. plantarum* and *L. rhamnosus* have been widely studied as probiotics [33,34], the probiotic potential of *L. senioris* and *L. curieae* identified here have rarely been reported. Therefore, the hemolytic activity assay was conducted firstly to ensure the safety of potential probiotic strains. Isolates that could cause partial or complete lysis of red blood cells were excluded. Only isolates that did not produce any zone around the colony were designated as no hemolysis. A total of 22 strains, 12 from *Bacillus*, were screened out to pass the hemolytic test and subjected to the following assay (Figure 1).

### 3.3. Determination of Gastrointestinal Survival and Biogenic Amine Formation

Survival in a gastrointestinal environment is a prerequisite for oral probiotic formulations to colonize the intestinal tract. Tolerance to artificial gastric juice and intestinal juice were tested among strains without hemolytic activity. A total of 18 out of 22 strains evaluated could survive through both gastric and intestinal juice (Figure 1).

The active metabolism of amino acids, usually decarboxylation in microorganisms, might result in high levels of biogenic amines, which would have negative effects on vasoactivity and psychoactivity, or as the precursors of carcinogenic nitrosamines [35]. Thus, biogenic amine formation is a very important evaluation index for the safety of probiotics. The biogenic amine formation by the isolates was determined using a screening assay for amino acid decarboxylation from ornithine and histidine. Ten isolates having strong biogenic amine formation were excluded through this assay. Representative results of strains without biogenic amine formation are displayed in Table 2.

Thus far, eight strains were screened out after the primary safety assessment and gastrointestinal tolerance evaluation. These strains were retained for subsequent characterization of probiotic properties. Three of them were *Lactobacillus* (N15-22, X16-68, X16-69), similar to *L. senioris* and *L. curieae*. The other five strains were Bacillales, among which two were *Bacillus*, similar to *B. licheniformis* (N17-02) and *B. ginsengihumi* (X16-66); the left three were assigned as *Bacillus* *megaterium* (N17-12), *Virgibacillus halophilus* (Y11-38) and *Paenibacillus chibensis* (Y13-51). Sequences of the 16S rRNA gene from these isolates were deposited in the GenBank, National Center of Biotechnology Information (NCBI) database under accession No. MW578436-MW578443 (accessed on 12 February 2021).

Overall, all of these eight strains had reasonable chance of survival in the simulated gastrointestinal environment, while Y11-38 and Y13-51 showed the most prominent adaptability (Table 3). Relatively higher survival rates were found in *Bacillus* strains than in *Lactobacillus* strains. The survival rates of *Bacillus* strains were in the range of 67.18–96.89% and rates of *Lactobacillus* strains were in the range of 3.60–25.00% after exposure to artificial gastric juice for 4 h. Similarly, in artificial intestinal juice they were 45.18–48.06% for *Bacillus* strains and 27.36–43.48% for *Lactobacillus* strains. This observation was consistent with previous reports about the greater viability of *Bacillus* strains than *Lactobacillus* strains when exposed to simulated gastrointestinal conditions or tea and coffee brewing [16,17,36].

### 3.4. Auto-Aggregation and Cell Surface Hydrophobicity

Probiotic candidates should be able to attach onto the epithelium lining of the ileum in the host before they could provide health benefits, thus their adhesion ability was assessed through auto-aggregation and cell surface hydrophobicity assay. High auto-aggregation is correlated with effective adhesion and subsequent colonization in the gastrointestinal tract. The auto-aggregation of these eight strains increased with the incubation time, reaching a range of 26.12–94.97% at 6 h and 52.53–95.56% at 24 h, respectively (Table 4). Among them, N15-22 and N17-02 had the highest auto-aggregation (95.56% and 93.69%) at 24 h. However, given that chyme spends on average 4–8 h in the ileum [18], *B. licheniformis* N17-02 was considered the best one due to the fact that its auto-aggregation rapidly increased in the first 6 h.

Hydrophobicity reveals the relative tendency of a substance to prefer nonaqueous rather than aqueous environments. A high cell surface hydrophobicity of a probiotic candidate indicates its probability to attach onto the epithelial lining of the intestine and resist the quick expelling from intestines. Here, the adhesion ability to hydrocarbons dichloromethane and ethyl-acetate were measured (Table 4). Interestingly, the hydrophobicity measurements in three *Lactobacillus* strains were higher than that of strains in Bacillales for both dichloromethane and ethyl-acetate, as high as 90.76–98.05% and 90.59–97.83%, respectively. Compared with the hydrophobicity reported previously, such as *Bacillus* strains to ethyl-acetate [21,29] and *Lactobacillus* isolates to dichloromethane [37], the hydrophobicity measurements of the strains evaluated here were comparable or even higher.

In general, based on the auto-aggregation and hydrophobicity, these eight strains have good adhesion ability. Probiotics that aggregate and accumulate in sufficient quantities to form an immune barrier could protect the gut from pathogens, maintain the balance and stability of intestinal flora and perform the other functions of probiotics [38].

### 3.5. Antibacterial Activity and Co-Aggregation

The antibacterial activity of isolates was tested against enteric bacterial pathogens, including *Staphylococcus aureus*, *Salmonella paratyphi*, *Escherichia coli* and *Bacillus subtilis* (Table 5). For Bacillales strains, significant inhibition was observed only in N17-02 against *S. aureus* and N17-12 against *S. paratyphi* and *E. coli*. The original supernatants of *Lactobacillus* strains culture had significant inhibitory effects against most pathogens tested except N15-22 against *E. coli*, but the antibacterial activity decreased after adjusting the supernatant to neutral. Thus, acid production might be an essential component of antibacterial activity in *Lactobacillus* strains, or acidic conditions might be beneficial for the function of their bacteriostatic substances. Similarly, organic acids including lactic acid were reported to be responsible for the antagonistic effect of several lactic acid bacteria derived from fermented food [39].

From the point of view of co-aggregation, these eight strains had co-aggregation at 46.67–69.18%, 50.98–67.49%, 48.00–59.36% and 70.78–82.10% to *S. aureus*, *S. paratyphi*, *E. coli* and *B. subtilis*, respectively (Table 5). These data implied that these isolates had good chances to co-aggregate with pathogens to interfere with their retention and colonization in gastrointestinal tract. The highest co-aggregation with *S. aureus* and *B. subtilis* was observed in X16-69 and N17-02, respectively. The highest co-aggregation with *S. paratyphi* and *E. coli* was found in N17-12, together with its significant antibacterial activity against these two pathogens, thus *B. megaterium* N17-12 was suggested as a good probiotic candidate from the point of view of the antibacterial potential. Three *B. megaterium* strains were isolated recently from stingless bee honey collected across Malaysia and subjected to probiotic screening, but they were found to have no inhibition effect against six pathogenic bacteria, including *Salmonella thyphimurium* and *E. coli* [40]. Thus, the probiotic property of *B. megaterium* was strain specific.

### 3.6. Antibiotic Susceptibility

Given the possibility of transfer of antibiotic resistance genes from probiotic bacteria to gut pathogens, the antibiotic susceptibility was evaluated in these eight strains. According to their classification and mechanisms of action [41], different antibiotics such as β-lactams (penicillin-G, ampicillin and cephalothin), macrolides (erythromycin), tetracyclines, aminoglycosides (gentamicin), glycopeptides (vancomycin) and chloramphenicol were chosen. Results indicated that each isolate was sensitive to at least three or more antibiotics (Table 6), which verified their safe utilization. Synthetically, Y13-51 showed the highest vulnerability to antibiotics tested. Most Bacillales strains were resistant to penicillin-G, probably due to the narrow antibacterial spectrum of penicillin-G. The *Lactobacillus* strains N15-22 and X16-68 were resistant to vancomycin and gentamicin, thus they might be applied to reduce the diarrhea caused by antibiotic damage to intestinal flora [42]. It needs to be further evaluated whether these antibiotic resistances were transferable before application.

At this point, eight isolates had met the selection criteria, i.e., gastrointestinal tract stress tolerance, adhesion ability, antimicrobial activity and primary safety assessment, to be probiotic candidates. Then, the cholesterol-lowering capabilities and carbon source utilization were further investigated to explore their functional properties.

### 3.7. Cholesterol-Lowering Potential

**Figure 2:**
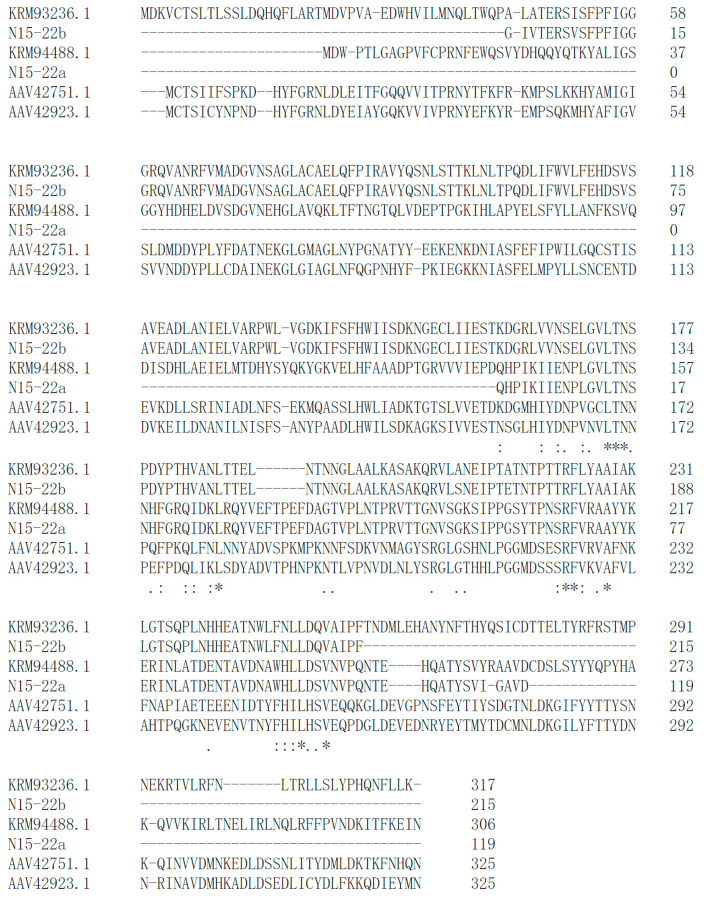
Amino acid sequence alignment of two putative BSHs, N15-22a and N15-22b, identified in N15-22 and the reference BSHs from NCBI. The closest reference BSHs were KRM94488.1 and KRM93236.1 from *Lactobacillus senioris* DSM 24302, while AAV42751.1 and AAV42923.1 were two functional BSHs reported from *Lactobacillus acidophilus* [45]. The identical amino acids are indicated by asterisks; the conservative and semi conservative amino acids are indicated by two dots and one dot, respectively. N in the LTN-conserved motif and R in the RF-conserved motif are the previously described conserved active-site residues.

Attention was first paid to the bile salt hydrolase (BSH), choloylglycine hydrolase (EC3.5.1.24), which is suggested to contribute to the detoxification of bile acid (BA), and is associated with the therapeutic effect of decreasing host cholesterol [19,43,44]. Unfortunately, using TDCA and GDCA as BA substrates, the positive BSH activity as a white precipitation zone around the colonies was not observed in our experiment. Since non-concordance between BSH phenotypic assay and presence of *bsh* gene was reported recently in *Enterococcus faecium* isolated from rhizosphere [30], we further searched for the *bsh* gene in these eight isolates. According to the *bsh* genes in similar strains as reference, touchdown PCR successfully retrieved six *bsh* genes from five isolates, including N17-02, N17-12, Y11-38, X16-66 and N15-22. In particular, two different *bsh* genes were identified in *L. senioris* N15-22 (Figure 2). High similarity was observed between the identified BSHs and the reference BSHs (Figure 2, Figure S2), which implied their functionality. A latest report in PNAS emphasized the substrate specificity of BSHs from *Lactobacillus*, which governs bacterial fitness and host colonization [45]. This might help explain the negative result in BSH activity assay, since only TDCA and GDCA were tested as substrates. Further analysis is needed to investigate whether these putative enzymes could act as functional BSHs.

**Figure 3:**
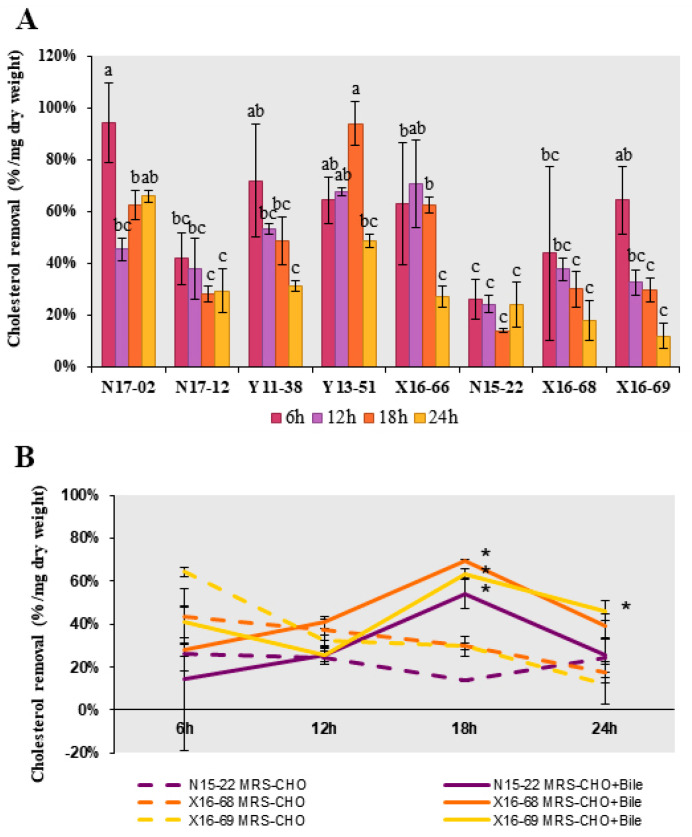
Cholesterol removal capability of the probiotic strains. (**A**). Cholesterol removal capability after incubation in cholesterol medium for 6 h, 12 h, 18 h and 24 h. Different superscripts (a–c) represent significantly different values (*p* < 0.05). (**B**). Cholesterol removal capability by *Lactobacillus* strains N15-22, X16-68, and X16-69 in MRS containing cholesterol (MRS-CHO) with or without bile salt. Values are expressed as mean ± SE (*n* = 3). The asterisk indicated the significantly different values (*p* < 0.05) between the mediums with or without bile salt for the same strain.

Then, the direct cholesterol removal capability, including cholesterol assimilation, absorption and adsorption, were evaluated in these eight strains. All of them displayed distinct cholesterol removal potential, and the removal rate ranged from 11.85% to 93.94% within 24 h (Figure 3A). The highest cholesterol removal rates were found over 90% at 6 h in *B. licheniformis* N17-02 and 18 h in *P. chibensis* Y13-51, respectively. Compared with three *Lactobacillus* strains, the removal rates were relatively higher in five isolates from Bacillales, ranging from 27.18% to 93.94%, which were higher than the removal rate 12.82–70.89% reported previously in *Bacillus* strains isolated from fermented vegetables [46]. To test whether bile salt could boost the relatively lower removal rate in *Lactobacillus* strains N15-22, X16-68 and X16-69, GDCA and TDCA were added. Significant increase of removal rate was observed at 18 h in all three strains, to a maximum of 69.48% in *L. curieae* X16-68 (Figure 3B). The addition of bile salts might affect the cell surface structure to varying degrees, thus promoting cholesterol removal rate [19].

Two possible mechanisms have been proposed regarding the hypocholesterolemia effect of probiotics: one is the direct binding or assimilation of cholesterol by probiotics and the other one is bile salt deconjugation catalyzed by BSH [19]. Intestinal bacterial BSHs cleave glycine or taurine from conjugated BAs, an essential upstream step for the production of deconjugated and secondary BAs. Deconjugated BAs are less soluble and resorbable than the conjugated ones in the intestine, thus rendering them easier to be excreted in feces and subsequently more conjugated BA should be synthesized de novo from cholesterol, thereby reducing the cholesterol concentration [19,47]. Another consequence is the lower emulsifying ability of deconjugated bile salts compared to conjugated ones, which may result in lower lipid digestion and decreased absorption of cholesterol, fatty acids and monoglycerides in the intestine [19,44,48]. The obvious cholesterol removal ability and the presence of *bsh* genes in these isolates here suggested that they could be good probiotic candidates with hypocholesterolemia effect. Especially, *B. licheniformis* N17-02 showed both remarkable cholesterol removal capability and presence of *bsh* gene.

BSH was confirmed to be the key factor affecting the hypocholesterolemia activity of *Lactobacillus*-fermented milk in hamsters [43] and *Lactobacillus-*yoghurt formulation in hypercholesterolemic adults [49]. Nevertheless, over-extensive secondary BA might be associated with diseases such as steatosis and colorectal carcinoma [19,50,51]. Therefore, more in vivo experiments are needed to support refined applications.

### 3.8. Carbohydrate Utilization Capability

The carbohydrate utilization profiles of these eight strains were investigated against two monosaccharides, three disaccharides, one trisaccharide, one tetrasaccharide and two sugar alcohols (Table 7). All eight strains could make use of at least five carbon sources. In particular, *B. megaterium* N17-12 showed significant growth on all the carbohydrate sources tested, including D-glucose, D-xylose, lactose, sucrose, cellobiose, stachyose, D-raffinose, sorbitol and mannitol. Such a wide carbohydrate utilization profile might suggest the growth advantage among GIT microbiota in host, where the competition in nutrition was fierce. Interestingly, N17-12 also displayed the strongest inhibitory effect against *S. paratyphi and E. coli* (Table 5), thus together confirming its great potential as probiotics. Other than being good as a probiotic to function in a host, the wide carbohydrate utilization profile also suggested its potential as a fermentation starter in fermented food or feed. For example, dough fermentation with *Lactobacillus* was investigated recently to improve the nutritional quality of foodstuff [52,53]. The application of these eight strains in the fermentation of food or feed will be investigated in the future.

## 4. Conclusions

In this study, probiotics screening and characterization of bacteria from PCR-Chachiensis was reported for the first time and results demonstrated that PCR-Chachiensis was a reservoir of potential probiotics. Our results indicated that eight isolates showed great survival in the simulated GIT stressful conditions, with nonhemolytic and no biogenic amine formation. Though these eight strains all displayed distinct cholesterol removal potential, *B. licheniformis* N17-02 showed both remarkable cholesterol removal capability and presence of the *bsh* gene, as well as possessing most of the desirable probiotic attributes. Thus, it could be a good probiotic candidate with hypocholesterolemic potential. *B. megaterium* N17-12 displayed the widest carbohydrate utilization profile and the strongest antimicrobial activity. Hence, it was promising to be used as a probiotic in host and as a fermentation starter in fermented food or feed. Further in vitro and in vivo analysis should be performed so that these isolates could be used for human or animal and food or feed applications.

## Figures and Tables

**Table 1:** Primers used to screen *bsh* gene.

Primers	Forward (5′–3′)	Reverse (5′–3′)
bsh N17-02	ACNATGGAYTTYGAYTTYGA	CNGGYTTCATRTGYTCYTT
bsh N17-12	AYTAYGAYTAYCAYCCNAA	YTCRTTCATNCCRTCCAT
bsh Y11-38	NGAYAARCARTGGAARAT	RNGCCATNCKNCCDATCAT
bsh Y13-51	GYCCNGGNATHACNGARGA	NACRCANARNCCRTGYTC
bsh X16-66	TAYGAYTTYGGNTAYATGC	YTCNARYTCYTCCCANAC
bsh N15-22-1	AYCARCAYCCNATHAARAT	TGRTANGGYTGRTARTARTA
bsh N15-22-2	GGCAYGTNATHYTNATGA	GTNCKYTTYTCRTTNGGC
bsh X16-68/69	ATHGGNGTNATGACNAA	GTNGTNGGNGTRTTNGT

**Table 2:** Representative results of strains passing the biogenic amine formation assay.

Strains	Medium	OD (590 nm)	pH	Chromogenic Reaction ^a^
N17-02	LB control	0.625 ± 0.009	7.31	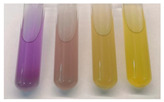
	LB-GL	0.365 ± 0.004	6.19	
	LB-GLH	0.175 ± 0.006	5.68	
	LB-GLO	0.126 ± 0.003	5.57	
N17-12	LB control	0.534 ± 0.015	6.9	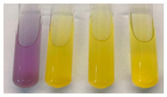
	LB-GL	0.052 ± 0.031	4.62	
	LB-GLH	0.032 ± 0.002	4.74	
	LB-GLO	0.030 ± 0.008	4.75	
Y11-38	LB control	0.616 ± 0.007	7.26	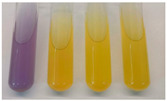
	LB-GL	0.045 ± 0.005	4.87	
	LB-GLH	0.037 ± 0.005	4.79	
	LB-GLO	0.049 ± 0.002	4.93	
Y13-51	LB control	0.457 ± 0.006	6.53	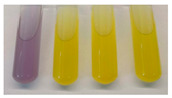
	LB-GL	0.038 ± 0.005	4.73	
	LB-GLH	0.037 ± 0.003	4.78	
	LB-GLO	0.036 ± 0.013	4.7	
X16-66	LB control	0.364 ± 0.006	6.21	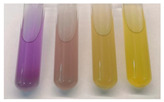
	LB-GL	0.062 ± 0.011	4.7	
	LB-GLH	0.064 ± 0.011	4.81	
	LB-GLO	0.065 ± 0.017	4.67	

^a^ Four test tubes from left to right indicating results for medium LB control, LB-GL, LB-GLH and LB-GLO, respectively.

**Table 3:** Relative survival ratio of strains in artificial gastrointestinal juice.

Strains	Gastric Juice (CFU/mL) × 10 ^4^		Percentage Survival (%)	Intestinal juice (CFU/mL) × 10 ^4^		Percentage Survival (%)
	Initial Count	Survival after 4 h		Initial Acount	Survival after 6 h	
N17-02	6.43 ± 0.30	6.23 ± 0.88	96.89 ± 9.33 ^a^	4.50 ± 1.62	2.03 ± 0.32	45.18 ± 9.26 ^a^
N17-12	4.83 ± 0.80	3.53 ± 0.24	73.10 ± 7.14 ^a^	1.06 ± 0.08	0.75 ± 0.10	70.98 ± 3.79 ^a^
Y11-38	2.33 ± 0.36	2.40 ± 0.25	103.00 ± 4.91 ^a^	1.87 ± 0.24	1.58 ± 0.14	84.31 ± 3.44 ^a^
Y13-51	3.33 ± 0.12	3.00 ± 0.58	90.00 ± 1.51 ^a^	1.57 ± 0.02	1.40 ± 0.04	89.36 ± 1.63 ^a^
X16-66	6.50 ± 0.21	4.37 ± 0.29	67.18 ± 2.32 ^a^	6.87 ± 0.79	3.30 ± 0.58	48.06 ± 3.02 ^a^
N15-22	3.70 ± 0.49	0.13 ± 0.04	3.60 ± 0.70 ^b^	9.20 ± 0.95	4.00 ± 1.00	43.48 ± 6.38 ^a^
X16-68	7.07 ± 0.64	1.77 ± 0.38	25.00 ± 3.19 ^b^	1.14 ± 0.32	0.58 ± 0.05	50.88 ± 9.76 ^a^
X16-69	2.00 ± 0.12	0.29 ± 0.08	14.48 ± 3.02 ^b^	1.10 ± 0.05	0.30 ± 0.08	27.36 ± 6.23 ^b^

Different superscripts (^a, b^) represent significantly different values in the same column (*p* < 0.05). Values are expressed as mean ± SE (*n* = 3).

**Table 4:** Auto-aggregation ability and hydrophobicity to ethyl-acetate and dichloromethane.

Strains	Auto-Aggregation Activity (%)				Hydrophobicity (%)	
	2 h	4 h	6 h	24 h	Ethyl-acetate	Dichloromethane
N17-02	64.87±1.14 ^a^	92.41±0.37 ^a^	94.97±1.12 ^a^	93.69±0.16 ^a^	10.32±6.11 ^d^	63.53±0.95 ^c^
N17-12	25.86±1.69 ^c^	36.31±0.46 ^d^	47.29±2.33 ^d^	80.24±1.98 ^b^	4.31±3.45 ^d^	40.02±1.62 ^d^
Y11-38	31.05±1.20 ^b^	54.59±1.31 ^b^	71.83±1.41 ^b^	77.89±0.97 ^b^	14.95±4.02 ^d^	26.89±0.74 ^e^
Y13-51	33.73±0.18 ^b^	36.89±2.13 ^d^	44.85±1.45 ^d^	52.53±0.98 ^e^	75.12±12.16 ^b^	98.93±0.55 ^a^
X16-66	13.29±1.92 ^d^	22.22±1.93 ^e^	27.18±0.33 ^f^	65.62±0.33 ^c^	53.56±1.13 ^c^	25.67±2.73 ^e^
N15-22	30.40±1.56 ^b^	45.87±2.00 ^c^	59.04±2.29 ^c^	95.56±0.65 ^a^	90.76±1.90 ^a^	96.88±1.28 ^a^
X16-68	24.38±2.39 ^c^	31.95±3.60 ^d^	39.43±3.45 ^e^	68.33±2.54 ^c^	98.05±0.42 ^a^	90.59±5.46 ^b^
X16-69	10.17±1.16 ^d^	15.51±0.57 ^f^	26.12±0.26 ^f^	57.67±2.21 ^d^	98.55±0.45 ^a^	97.83±1.64 ^a^

Different superscripts (^a, b, c, d, e, f,^) represent significantly different values (*p* < 0.05) in the same column. Values are expressed as mean ± SE (*n* = 3).

**Table 5:** Antibacterial activity and co-aggregation ability of probiotic strains against indicator strains.

Strains	Antibacterial Activity ^A^ and Co-Aggregation (%) ^B^							
	*Staphylococcus aureus*		*Salmonella paratyphi*		*Escherichia coli*		*Bacillus subtilis*	
N17-02	+++	50.66 ± 1.57 ^e^	+	54.93 ± 1.23 ^c^	+	51.99 ± 8.94 ^b^	+	82.10 ± 0.89 ^a^
N17-12	-	55.26 ± 2.90 ^d^	++	67.49 ± 3.30 ^a^	+++	59.36 ± 1.12 ^a^	-	81.28 ± 1.07 ^a^
Y11-38	+	52.36 ± 1.30 ^d^	-	57.23 ± 2.41 ^b^	+	53.09 ± 3.19 ^a^	-	72.05 ± 1.36 ^c^
Y13-51	+	46.74 ± 0.59 ^f^	-	52.55 ± 0.42 ^c^	+	50.51 ± 1.91 ^b^	-	73.12 ± 2.10 ^c^
X16-66	+	56.55 ± 1.91 ^c^	-	59.64 ± 1.67 ^b^	-	56.92 ± 3.03 ^a^	-	77.45 ± 1.36 ^b^
N15-22	+++/+ ^C^	53.56 ± 0.73 ^d^	+++/+	53.84 ± 1.36 ^c^	+/-	48.00 ± 1.85 ^b^	+++/+	81.47 ± 0.86 ^a^
X16-68	+++/+	58.70 ± 3.19 ^b^	+++/+	50.98 ± 1.68 ^d^	+++/+	55.08 ± 1.13 ^a^	+++/+	73.94 ± 1.30 ^c^
X16-69	+++/+	69.18 ± 0.39 ^a^	+++/+	51.32 ± 2.53 ^d^	++/+	56.29 ± 2.24 ^a^	+++/+	70.78 ± 0.57 ^d^

^A^ The plus or minus sign indicates the difference in antibacterial activity among probiotics strains. -, no antibacterial activity; +, antibacterial activity but not significant; ++, significant antibacterial activity as *p* < 0.05; +++, significant antibacterial activity as *p* < 0.01. ^B^ Co-aggregation is expressed as percentage. ^C^ The plus or minus sign before and after slash indicate antibacterial activity using original supernatant in its initial acidic pH and supernatant neutralized to pH 6.5. Different lowercase superscripts (^a, b, c, d, e, f^) represent significantly different values (*p* < 0.05) in the same column. Values are expressed as mean ± SE (*n* = 3).

**Table 6:** Antibiotic susceptibility of probiotic strains.

Antibiotics	Strains							
	N17-02	N17-12	Y11-38	Y13-51	X16-66	N15-22	X16-69	X16-68
Penicillin-G (10 µg/disc)	R	R	R	MS	S	MS	S	S
Ampicillin (10 µg/disc)	R	S	S	S	S	S	S	S
Cephalothin (30 µg/disc)	R	S	MS	S	S	S	S	S
Erythromycin (15 µg/disc)	S	S	S	S	S	S	S	S
Tetracycline (30 µg/disc)	S	S	S	S	S	R	S	S
Gentamicin (10 ug/disc)	S	S	S	S	S	R	MS	R
Vancomycin (30 µg/disc)	MS	MS	S	S	R	R	MS	R
Chloramphenicol (30 ug/disc)	R	S	S	S	S	S	S	S

R, resistant; S, sensitive; MS, moderately sensitive.

**Table 7:** The utilization of different carbon source by probiotic strains.

Carbon source	Strains							
	N17-02	N17-12	Y11-38	Y13-51	X16-66	N15-22	X16-68	X16-69
D-Glucose	+	++	+	+	-	+	+	+
D-Xylose	-	++	-	++	-	+	+	+
Lactose	-	++	+	+	+	-	-	+
Sucrose	+	++	-	-	+	+	+	+
Cellobiose	+	++	+	-	-	+	+	+
Stachyose	+	++	+	-	+	+	+	+
D-Raffinose	+	++	++	+	+	+	+	+
Sorbitol	-	++	+	+	-	+	-	+
Mannitol	+	++	+	-	+	+	-	+

The plus or minus sign indicates the difference in the utilization degree of carbon source in different strains. -, unusable; +, usable but not significant; ++, significant utilization (*p* < 0.05).

## Data Availability

Not applicable.

## Supplementary Materials

The following are available online at https://www.mdpi.com/article/10.3390/microorganisms9061224/s1, Figure S1: *Pericarpium Citri Reticulatae* ‘Chachiensis’ (PCR-Chachiensis, in Chinese *Guang Chen Pi*), is the aged pericarps of Citri Reticulatae Blanco cv. Chachiensis. Figure S2: Partial amino acid sequence alignment of the BSHs encoding by *bsh* genes detected in probiotic strains and their reference BSHs from NCBI. Table S1: Detail information of 64 isolated strains.
