# Supplementary material for: Probiotic Potential and Cholesterol-Lowering Capabilities of Bacterial Strains Isolated from Pericarpium Citri Reticulatae ‘Chachiensis’
Source: Microorganisms. 2021 Jun 4;9(6):1224. doi: 10.3390/microorganisms9061224 (PMC8227569; doi:10.3390/microorganisms9061224)
Supplement: Supplementary file 1 [file microorganisms-09-01224-s001.zip › microorganisms-1244369-supplementary.pdf]

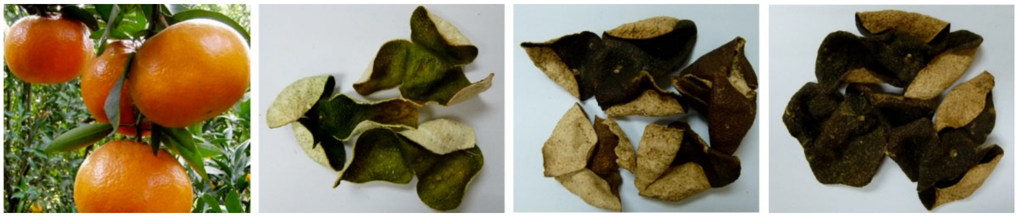

**Figure S1.** *Pericarpium Citri Reticulatae* 'Chachiensis' (PCR-Chachiensis, in Chinese *Guang Chen Pi*), is the aged pericarps of Citri Reticulatae Blanco cv. Chachiensis.

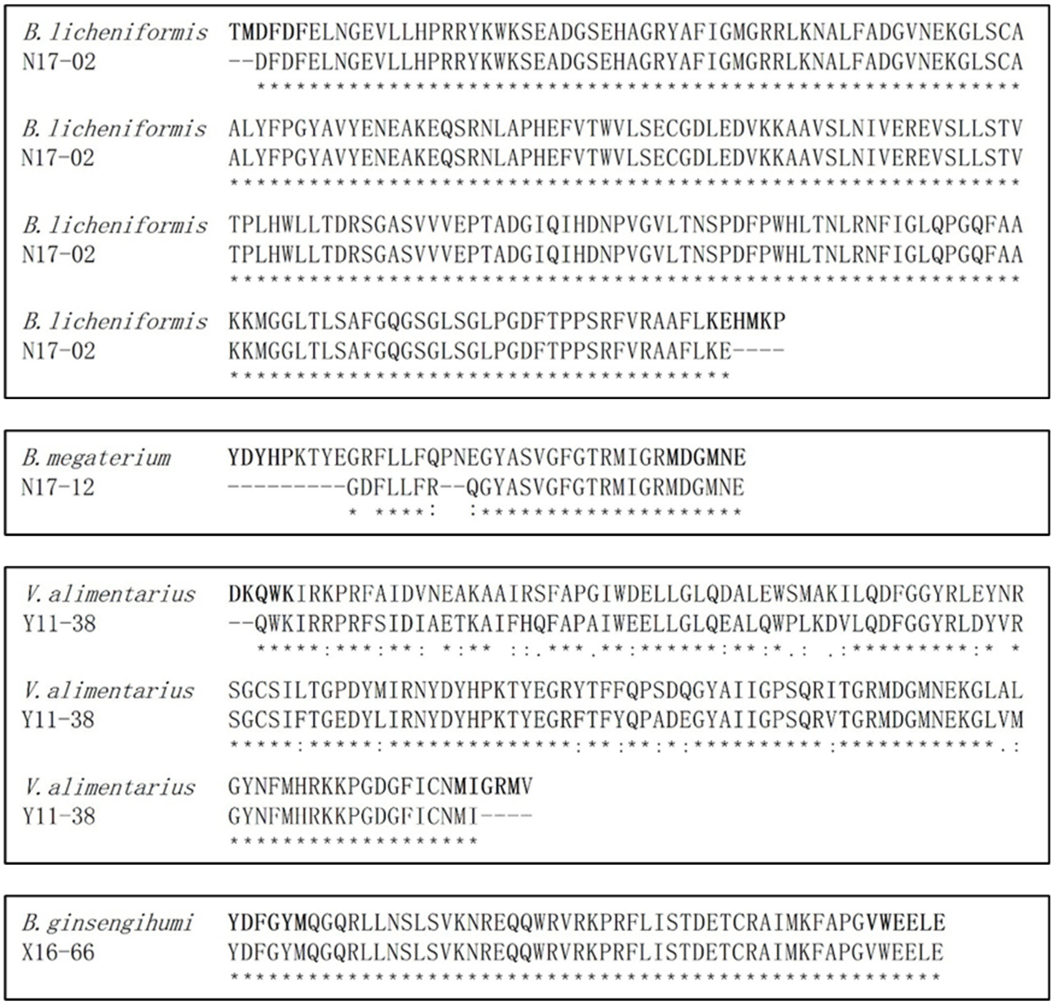


**Figure S2.** Partial amino acid sequence alignment of the BSHs encoding by *bsh* genes detected in probiotic strains and their reference BSHs from NCBI. The protein ID of the reference BSHs in NCBI were WP_094023840.1 (*Bacillus licheniformis*), WP_116516373.1 (*Bacillus megaterium*), WP_029268033.1 (*Virgibacillus alimentarius*), WP_025726634.1 (*Bacillus ginsengihumi*). The identical amino acids are indicated by asterisks; the conservative and semi conservative amino acids are indicated by two dots and one dot respectively.

**Table S1.** Detail information of 64 isolated strains.

| Strains | Hit taxon name | Identity (%) | 16S rDNA accession No. of hit strain | Culture media | Hemolytic | Gastrointestinal survival | Amine production |
|---|---|---|---|---|---|---|---|
| N17-01 | *Bacillus cereus* | 97.97% | MK389424.1 | Elliker | + | N/A | N/A |
| N17-02 | *Bacillus licheniformis* | 99.93% | KU877628.1 | Elliker | - | + | - |
| N17-03 | *Bacillus tequilensis* | 100% | MF077125.1 | Elliker | + | N/A | N/A |
| N17-04 | *Bacillus aryabhattai* | 99.93% | KR063195.1 | Elliker | - | - | N/A |
| N17-05 | *Bacillus cereus* | 100% | MG309432.1 | Elliker | + | N/A | N/A |
| N17-06 | *Bacillus cereus* | 100% | KY316446.1 | Elliker | + | N/A | N/A |
| N16-07 | *Bacillus pumilus* | 100% | KF158227.1 | Elliker | + | N/A | N/A |
| N17-08 | *Bacillus licheniformis* | 99.93% | MH000674.1 | Elliker | - | + | + |
| N16-09 | *Rummeliibacillus stabekisii* | 100% | MT658588.1 | Elliker | + | N/A | N/A |

| | | | | | | | |
|---|---|---|---|---|---|---|---|
| N17-10 | *Bacillus velezensis* | 98.95% | MH261042.1 | LB | + | N/A | N/A |
| N17-11 | *Bacillus aryabhattai* | 99.79% | KR063195.1 | LB | + | N/A | N/A |
| N17-12 | *Bacillus megaterium* | 99.93% | MK521052.1 | LB | - | + | - |
| N16-13 | *Bacillus firmus* | 99.85% | FJ613310.1 | LB | + | N/A | N/A |
| N16-14 | *Bacillus aquimaris* | 100% | EU835730.1 | LB | + | N/A | N/A |
| N16-16 | *Bacillus altitudinis* | 100% | MN710447.1 | LB | + | N/A | N/A |
| N16-17 | *Paenibacillus illinoisensis* | 99.58% | NR_113828.1 | LB | + | N/A | N/A |
| N16-18 | *Bacillus sporothermodurans* | 100% | MN904954.1 | LB | + | N/A | N/A |
| N15-19 | *Bacillus amyloliquefaciens* | 100% | GQ853414.1 | LB | + | N/A | N/A |
| N15-20 | *Bacillus subtilis* | 100% | MH100679.1 | LB | - | + | + |
| N15-21 | *Quasibacillus thermotolerans* | 100% | MG651240.1 | Elliker | - | + | + |
| N15-22 | *Lactobacillus senioris* | 100% | MW578443.1 | MRS | - | + | - |
| N16-23 | *Bhargavaea cecembensis* | 99.86% | JQ071510.1 | Elliker | + | N/A | N/A |
| N16-24 | *Terribacillus saccharophilus* | 99.93% | NR_041356.1 | Elliker | + | N/A | N/A |
| N16-25 | *Paenibacillus lautus* | 97.16% | MT510152.1 | LB | + | N/A | N/A |
| Y13-26 | *Bacillus tequilensis* | 100% | JF411301.1 | Elliker | + | N/A | N/A |
| Y13-27 | *Bacillus cereus* | 100% | MF662458.1 | Elliker | + | N/A | N/A |
| Y13-28 | *Bacillus kochii* | 99.79% | MN075515.1 | Elliker | + | N/A | N/A |
| Y13-29 | *Bacillus licheniformis* | 96.58% | MH261078.1 | Elliker | + | N/A | N/A |
| Y12-30 | *Bacillus amyloliquefaciens* | 100% | KY685066.1 | Elliker | + | N/A | N/A |
| Y11-31 | *Bacillus altitudinis* | 100% | KJ534473.1 | Elliker | + | N/A | N/A |
| Y11-32 | *Bacillus thuringiensis* | 99.86% | MN594796.1 | Elliker | + | N/A | N/A |
| Y13-33 | *Lysinibacillus pakistanensis* | 95.03% | MN396729.1 | LB | - | + | + |
| Y12-34 | *Bacillus licheniformis* | 100% | MN746177.1 | LB | + | N/A | N/A |
| Y12-35 | *Bacillus pumilus* | 99.86% | FJ237277.1 | LB | + | N/A | N/A |
| Y11-36 | *Bacillus safensis* | 100% | HQ699512.1 | LB | + | N/A | N/A |
| Y11-37 | *Bacillus coagulans* | 96.77% | MT509777.1 | LB | + | N/A | N/A |
| Y11-38 | *Virgibacillus halophilus* | 99.59% | NR_041358.1 | LB | - | + | - |
| Y13-39 | *Bacillus circulans* | 99.79% | KM349200.1 | LB | - | - | N/A |
| Y13-40 | *Sporosarcina contaminans* | 99.65% | NR_116955.1 | LB | - | - | N/A |
| Y11-41 | *Bacillus aryabhattai* | 99.86% | KC764988.1 | Elliker | + | N/A | N/A |
| Y11-42 | *Bacillus megaterium* | 99.93% | JF683607.1 | Elliker | + | N/A | N/A |
| Y11-43 | *Bacillus megaterium* | 100% | MF431758.1 | Elliker | - | - | N/A |
| Y11-45 | *Bacillus oleronius* | 99.79% | LC588627.1 | Elliker | + | N/A | N/A |
| Y12-47 | *Bacillus cereus* | 99.86% | JN400121.1 | Elliker | + | N/A | N/A |
| Y12-48 | *Bacillus coagulans* | 99.86% | MT505650.1 | Elliker | + | N/A | N/A |
| Y11-49 | *Bacillus altitudinis* | 100% | KJ534473.1 | Elliker | + | N/A | N/A |
| Y11-50 | *Paenibacillus lautus* | 99.72% | LC588571.1 | LB | - | + | + |
| Y13-51 | *Paenibacillus chibensis* | 98.27% | MN421017.1 | LB | - | + | - |
| Y11-53 | *Bacillus coagulans* | 96.62% | MK685115.1 | MRS | + | N/A | N/A |
| Y11-54 | *Bacillus coagulans* | 97.50% | MH685469.1 | MRS | + | N/A | N/A |
| Y11-55 | *Bacillus coagulans* | 94.97% | MH685469.1 | MRS | + | N/A | N/A |
| Y11-57 | *Bacillus circulans* | 98.89% | FJ581445.1 | Elliker | + | N/A | N/A |
| X15-58 | *Bacillus velezensis* | 100% | MT611666.1 | LB | - | + | + |
| X15-59 | *Bacillus subtilis* | 100% | KX960108.1 | LB | + | N/A | N/A |
| X15-60 | *Bacillus velezensis* | 99.79% | MN704466.1 | LB | + | N/A | N/A |
| X14-61 | *Paenibacillus chitinolyticus* | 98.47% | MN704459.1 | LB | - | + | + |
| X14-62 | *Bacillus licheniformis* | 96.53% | KX784914.1 | LB | - | N/A | N/A |
| X15-63 | *Bacillus oleronius* | 99.93% | KY773585.1 | LB | - | + | + |
| X15-64 | *Bacillus licheniformis* | 99.65% | MH475938.1 | LB | - | + | + |
| X14-65 | *Bacillus tequilensis* | 100% | MF077125.1 | LB | + | N/A | N/A |
| X16-66 | *Bacillus ginsengihumi* | 99.86% | NR_041378.1 | Elliker | - | + | - |
| X14-67 | *Bacillus pumilus* | 100% | MN581175.1 | LB | + | N/A | N/A |
| X16-68 | *Lactobacillus curieae* | 95.40% | LC521989.1 | MRS | - | + | - |
| X16-69 | *Lactobacillus curieae* | 95.49% | LC519995.1 | MRS | - | + | - |

The plus sign indicates that the strain has this ability, while the minus sign indicates the opposite. N/A indicated that the strain was not tested in this term.
